# Linguistically informed ChatGPT prompts to enhance Japanese-Chinese machine translation: A case study on attributive clauses

**DOI:** 10.1371/journal.pone.0313264

**Published:** 2025-01-09

**Authors:** Wenshi Gu

**Affiliations:** School of Foreign Languages, Beihang University, Beijing, China; Shanghai International Studies University - Songjiang Campus, CHINA

## Abstract

In the field of Japanese-Chinese translation linguistics, the issue of correctly translating attributive clauses has persistently proven to be challenging. Present-day machine translation tools often fail to accurately translate attributive clauses from Japanese to Chinese. In light of this, this paper investigates the linguistic problem underlying such difficulties, namely how does the semantic role of the modified noun affect the selection of translation patterns for attributive clauses, from a linguistic perspective. Through the analysis of numerous examples, the study develops a novel three-step prompt chaining strategy, which was tested using ChatGPT. The experimental results demonstrate that this approach significantly improves translation quality, with an average score increase of over 43%. These findings highlight the effectiveness and potential of linguistically informed prompt design in enhancing the translation accuracy of complex sentence structures. This study not only offers a new perspective on the integration of linguistics theory and machine translation technologies, but also provides valuable insights for optimizing large language models prompt and improving language education tools.

## 1: Introduction

The development of machine translation (MT) has garnered significant attention in the industry. Essentially a complex task, machine translation involves transforming a source input into semantically equivalent target output in a different language, requiring both sequence understanding and generation [1]. Among the most recent and promising approaches to MT is neural machine translation (NMT), which utilizes neural networks and handles massive datasets to learn from them [2]. NMT is capable of generating more natural and fluent translations than its predecessors and is presently utilized in various commercial products such as Google Translation [3, 4], and Baidu Translation [5].

While NMT theoretically achieves high-quality output for high-resource languages [6], translation errors persist due to the lack of domain-specific training data [7]. For example, in Chinese-English translation, MT systems often overlook or misinterpret metadiscourse, leading to issues with textual coherence and reader engagement [8]. Similar challenges arise in Japanese-Chinese translation. Commercial NMT platforms like Google Translate and Baidu Translate struggle when translating complex Japanese sentences, particularly those with long attributive clauses. These platforms tend to directly translate Japanese long attributive clauses into similarly structured Chinese clauses, which are often unnatural for native Chinese readers. Furthermore, translation quality is inconsistent, with the same Japanese attributive clause structures being translated in different ways, leading to variable results.

For instance, when using commercial MT platforms, the "inner relation" attributive clause in (1a) is translated as (1b), while (2a) and (3a) are rendered as (2b) and (3b), respectively. In the source language (SL) and target language (TL), single underlining indicates the attributive clause, and double underlining highlights the modified noun. Translations marked with "?" indicate unnatural or awkward Chinese renderings.

(1a)SL:どちらかというと白羽さんが性犯罪者寸前の人間だと思っていたので、迷惑をかけられたアルバイト女性や女性客のことも考えずに、自分の苦しみの比喩として気軽に強姦という言葉を使う白羽さんを、被害者意識は強いのに、自分が加害者かもしれないとは考えない思考回路なんだなあ、と思って眺めた。(I considered him one step short of being a sex offender, but here he was casually likening his own suffering to sexual assault without sparing a thought for all the trouble he’d caused for women store workers and customers. He seemed to have this odd circuitry in his mind that allowed him to see himself only as the victim and never the perpetrator I thought as I watched him.)

(コンビニ人間 [9] (CONVENIENCE STORE WOMAN [10]))

(1b)TL:? 以为白羽先生快要成为性侵者了, 所以没想到打工的女性和被打扰的女性顾客, 随口说出我被强奸来比喻自己的苦难我看着用词的白羽先生, 心想他虽然有很强的受害意识, 却不认为自己可能是肇事者。

(Google Translation 20230316)

(2a) SL: 掌やポケットの中で小銭を鳴らしている人は、煙草か新聞をさっと買って帰ろうとしている人が多いので、お金の音には敏感だ。(It’s a sound I’m sensitive to, since customers who come just to buy cigarettes or a newspaper often jingle coins in their hand or pocket.)

(コンビニ人間 (CONVENIENCE STORE WOMAN))

(2b) ?手掌或口袋里有硬币叮当作响的人应该赶紧去买香烟或报纸。很多人都想着回家, 所以对金钱的声音很敏感。

(Google Translation 202401007)

(3a) SL: 再びおにぎりを並べに走ろうとした私に、バイトリーダーの泉さんが声をかける。(I am just running to put out more rice balls when our supervisor, Mrs. Izumi, calls out to me.)

(コンビニ人間 (CONVENIENCE STORE WOMAN))

(3b) TL:当我正要跑去重新排饭团时, 兼职组长泉小姐叫住了我。

(Google Translation 202401007)

In example (1a), there is an extremely long attributive clause "迷惑をかけられたアルバイト女性や女性客のことも考えずに、自分の苦しみの比喩として気軽に強姦という言葉を使う(he was casually likening his own suffering to sexual assault without sparing a thought for all the trouble he’d caused for women store workers and customers)"in front of the noun "白羽さん(Mr. Shiraha) ". The translation website struggles to directly translate Japanese "attributive clause + modified noun" structures, such as (1a), into Chinese "attributive clause + modified noun" structures ((1b)), leading to comprehension difficulties for Chinese native speakers. While translation (2b) also maintains the original Japanese attributive clause structure, resulting in an unnatural Chinese sentence, translation (3b) adopts a more idiomatic Chinese expression to convey the same meaning.

(1b) and (2b) show that NMT platforms tend to translate Japanese "attributive clause + modified noun" structures directly into Chinese, resulting in unnatural translations. The contrast between (2b) and (3b) demonstrates how NMT platforms may apply inconsistent translation strategies to similar sentences, affecting the quality of the output.

Given that NMT platforms underperform when handling the "attributive clause + modified noun" structure in Japanese-Chinese translation, the question arises: How effective are newer large language models (LLMs) in addressing this challenge?

In 2022, OpenAI introduced ChatGPT [11], a large language model that is derived from the InstructGPT architecture [12]. The model is trained to follow specific prompts and deliver detailed responses. According to the official release, ChatGPT can address follow-up questions, identify and rectify erroneous responses, challenge false assumptions, and decline inappropriate requests, among other features, thanks to its conversational format. Its various natural language processing abilities include code debugging, logic reasoning, storytelling, machine translation, question answering, and more. These capabilities, along with subsequent LLMs like Google Gemini [13], have shown impressive effectiveness in foreign language learning, education, and writing [14–16].

Furthermore, LLMs like ChatGPT, which are pretrained on massive, unlabelled corpora, have displayed remarkable emergent abilities when subjected to model scaling. This allows for prompt-based downstream applications instead of task-specific fine-tuning [17–19]. Prompting, which involves rephrasing test samples with descriptive task instructions and then feeding these prompts directly to LLMs, is being actively explored.

This leads to the question: Can LLMs like ChatGPT resolve the issues that NMT platforms face in Japanese-Chinese translation tasks? As seen in (4a) and (4b), ChatGPT continues to struggle with translating the Japanese "attributive clause + modified noun" structure directly into Chinese, resulting in translations that are less natural and fluent. This reduces both the accuracy and readability of the target text. A comparison between (4b) and (5b) reveals that ChatGPT applies different translation methods for similar Japanese structures, leading to inconsistent quality.

(4a) SL: 駅前のショッピングモールで買ったケーキを食べながら、皆の顔を見て懐かしい懐かしいと連呼するユカリに皆が笑った。(We all laughed as she kept looking around, saying how much she’d missed us as we nibbled at the cakes from the station mall.)

(コンビニ人間 (CONVENIENCE STORE WOMAN))

(4b) ?一边吃着在车站前购买的蛋糕, 一边看着大家的脸, 不停地喊着“好怀念, 好怀念”的Yukari让大家都笑了。

(ChatGPT3.5 20230326)

(5a) SL:社員の真似をして、勢いよくお辞儀をした私に、女性は笑って「ありがとうね、またきます」と言い、レジから去って行った。(’Thank you!’ I said, enthusiastically bowing the way the manager had done. The woman laughed and said, ’Thank you, I’ll come again, ’ and moved away from the till.)

(コンビニ人間 (CONVENIENCE STORE WOMAN))

(5b) 看到店员的样子, 我也效仿着热情地鞠了一躬, 然后那位女性笑着对我说:“谢谢啦, 下次还会再来的。”她说完后离开了收银台。

(ChatGPT3.5 20230326)

The "attributive clause + modified noun" structure is common in Japanese. However, only about 50% of these structures can be directly translated into equivalent "attributive clause + modified noun" structures in Chinese [20]. Identifying the proper translation patterns for such structures has long been a central focus in Japanese-Chinese translation studies.

Despite the limitations of NMT and LLMs in handling the translation of "attributive clause + modified noun" structures, no research to date has provided effective, linguistically informed solutions to improve the quality of LLM-generated translations.

This study aims to leverage the emergent capabilities of LLMs to address the following key questions:

1. Can linguistics theory be applied to design prompts that enhance the quality of Chinese translations of Japanese sentences with complex attributive clause structures?
2. Can linguistics theory be used to design prompts that improve the robustness and consistency of the translation methods chosen by LLMs when translating Japanese sentences with complex attributive clause structures?

These are the main research objectives of this paper, which aims to address gaps in the existing literature and provide a fresh perspective on the application of prompt optimization techniques in machine translation.

## 2: Related work

### 2.1: Literature review: Translation methods for Japanese attributive clauses

[21] proposed two translation methods for Japanese attributive clauses. Method A involves directly rendering shorter attributive clauses as attributive structures in Chinese (the original text"短的定语可以直接照译"). Method B is applied to longer attributive clauses, where it is often necessary to relocate the attributive clause to the end of the main sentence (the original text"较长的递加定语多数要后移"). Additionally, [21] proposed four specific translation methods for longer Japanese attributive clauses as mentioned in Method B. Method B-1: Translate the attributive clause into Chinese directly without changing the modification relationship (the original text"不变语序, 直接连译"). Method B-2: Translate the attributive clause into a separate sentence, followed by the main clause(the original text"定语提位, 代指下连"). Method B-3: Translate the main clause first and then translate the attributive clause(the original text"先抓住干, 后理分支"). Method B-4: Divide the attributive clause into two parts, use one part as a modifier before the modified noun, and translate the other part as a complement(the original text"部分定语不变, 部分定语后置").

[22] proposed three translation methods for translating longer Japanese attributive clauses. Method C: Long Japanese attributive clauses can be translated into long attributive clauses in Chinese(the original text"長い連体修飾語をそのまま訳して中国語でも長い連体修飾語とする"). Method D: Use long attributive clauses as predicates, with the center words serving as subjects, and then continue the sentence with appropriate words(the original text"長い連体修飾語を述語とし、その中心語を主語として、独立したセンテンスにする。そして、そのあとに適当なことばでそれを受けて話をつづける"). Method E: Position the center word first (or along with a short attributive clause) and process the long attributive clause as a supplementary explanation that complements the center word with appropriate words (which may not always be necessary) (the original text"中心語、またはプラス短い連体修飾語をさきに出し、長い連体修飾語は適当な言葉で、使わない場合もあるが、中心語を受けてそれを補足説明する形として処理する").

Although [21, 22] proposed translation methods for Japanese attributive clauses, they did not elaborate on the characteristics of the "attributive clause + modified noun" structure in Japanese that each method is best suited for, nor did they discuss whether the semantic role of the modified noun influences the selection of a translation method. This lack of detail limits the development of appropriate prompts for LLMs based on these methods.

### 2.2: Large language model and prompting for machine translation

There are substantial differences between the output processes of LLMs and NMT.

**Fig 1:**
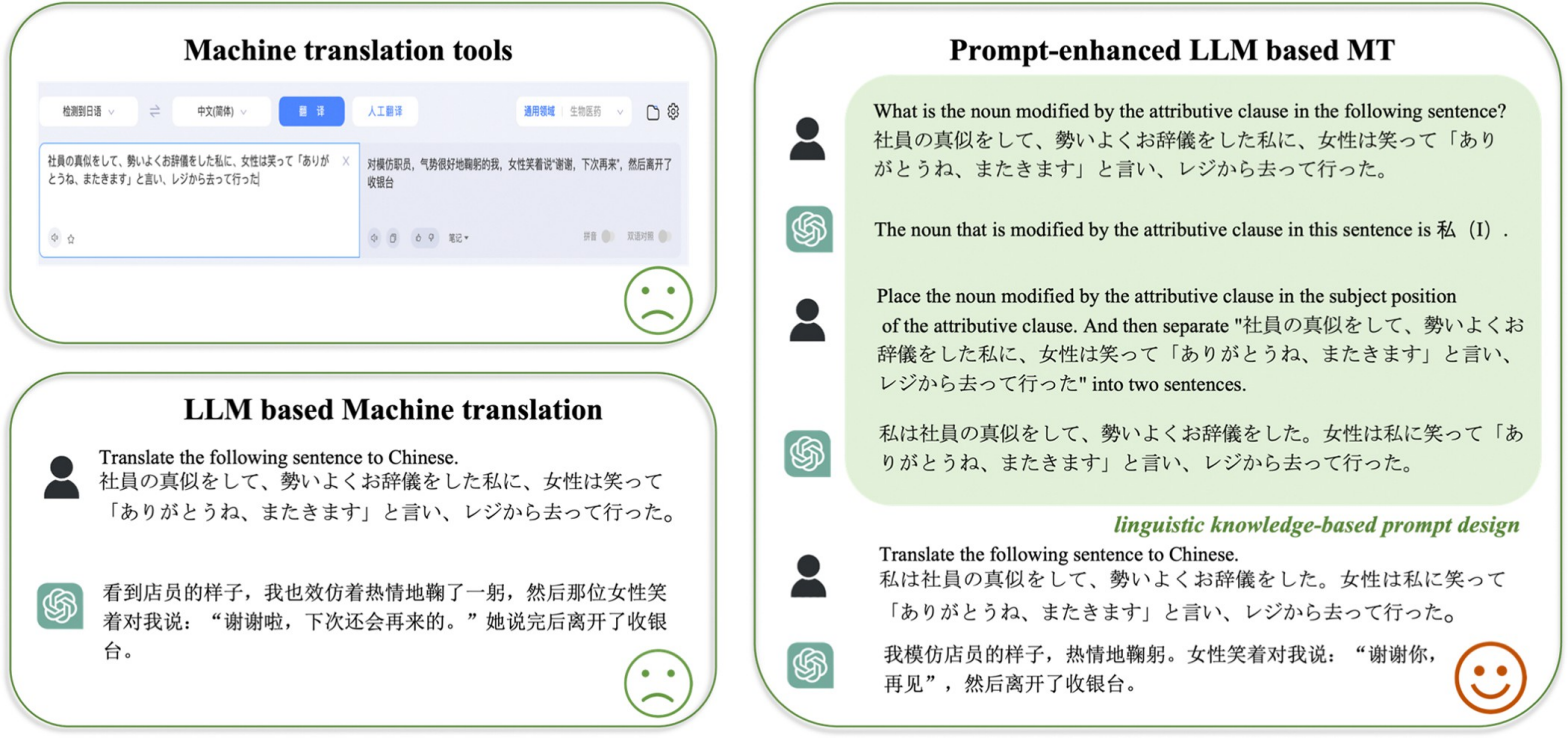
Illustration of our proposed prompt-enhanced MT framework compared to conventional NMT tools (e.g. **Baidu translation) and vanilla LLM-based MT.** Note: The machine translation site utilized in this study generated all translated texts, which were obtained on March 26, 2023.

Fig 1 illustrates that conventional NMT tools directly present the translated version of the original input sentence. In contrast, LLMs require a specific template to be applied for formatting the original sentence to generate the translation, such as, ’Translate the following sentence to Chinese: {original input sentence}’. In other words, given a pretrained and fixed LLMs, MT prompting first converts each test input to a prompt according to a template and then generates the translation by feeding the prompt to the LLMs. This highlights that the quality of the output from large language models is largely dependent on the quality of the prompts [15]. Therefore, by optimizing the design of prompts, we can significantly enhance the output quality of LLMs-based translations [23].

The central focus of this paper is to explore how the learning capabilities of large language models can be leveraged to incorporate linguistics theory into prompt design, thereby guiding these models to produce translations that better meet user expectations.

## 3: Research subjects

To minimize the influence of confounding factors and ensure the reliability of experimental results, this study focuses on a specific type of Japanese attributive clause: "Inner relation" attributive clauses, where the semantic relationship between the modified noun and the main verb of the clause is nominative case.

The scoped object of this paper is an attributive clause that satisfies the following three conditions.

- "Inner relation" Japanese attributive clause.
- The predicate in the main clause is a verb or a verb phrase.
- The nominative case is the only valid semantic role for the modified noun in the attributive clause under consideration.

According to (a), this paper does not focus on the "Outer relation" Japanese attributive clause, such as the one in (6). Additionally, as stated in (b) and (c), examples like (7) and (8), even if they are "Inner relation" Japanese attributive clauses, are excluded from the scope of this research. Therefore, only cases like (9), where the predicate in the main clause is a verb or verb phrase and the modified noun assumes a nominative semantic role in the attributive clause, are considered as the research objects.

(6)太郎がテレビを壊した可能性がある。 (There is a possibility that Taro broke the TV.)

(Created by the author)

(7)太郎が壊したテレビは高い。(The TV that Taro broke is expensive.)

(Created by the author)

(8)太郎が買ったテレビは壊れた。(The TV that Taro bought is broken.)

(Created by the author)

(9)テレビを壊した太郎は頭を下げた。(Taro who broke the TV bowed his head.)

(Created by the author)

This paper refers to Japanese attributive clauses that satisfy the three conditions (a), (b), and (c) simultaneously, such as the example sentence (9), as "nominative" inner relation attributive clauses.

## 4: Methodology

This study explores how linguistics theory can be utilized to optimize prompt design, with the goal of improving the translation quality of large LLMs when handling Japanese sentences containing an "attributive clause + modified noun" structure. To accomplish this, the paper proposes a novel prompt design approach, focusing on the semantic role of the modified noun and analyzing how this influences the selection of translation methods used for these Japanese structures when translating into Chinese. The linguistic conclusions derived from this analysis are incorporated into a general prompt design, and the effectiveness of this approach is tested by comparing the translation quality of zero-shot prompts with that of optimized prompt combinations.

### Data selection and collection

The study compiled a parallel corpus consisting of five officially published modern Japanese novels and their corresponding Chinese translations. The selected novels include Half a Confession (《半落ち》 [24]), Convenience Store Woman (《コンビニ人間》), Secret (《秘密》 [25]), Wings in the Darkness (《翼のある闇》 [26]), and The Housekeeper and the Professor (《博士の愛した数式》 [27]). Their respective Chinese translations are Half a Confession (《半落》 [28]), Convenience Store Woman (《人间便利店》 [29]), Secret (《秘密》 [30]), Wings in the Darkness (《有翼之暗》 [31]), and The Housekeeper and the Professor (《博士的爱情算式》 [32]). These novels span a range of genres, including suspense, ethics, and science fiction, with language that closely mirrors everyday speech, ensuring that the corpus is both accurate and rich in linguistic diversity.

Through manual annotation, 64 sentence pairs featuring a "nominative inner relation attributive clause + modified noun" structure was extracted from the parallel corpus, along with their corresponding Chinese translations. Dialectal variations were excluded during data selection to enhance the generalizability of the study’s findings.

### Potential biases

To mitigate potential biases associated with using a single source of data, the study included a diverse range of materials covering different themes. However, it is important to note that the linguistic expressions found in literary works may differ from those used in everyday language, which could influence the evaluation of translation quality.

### Statistical methodology

To provide a clear and concise representation of how prompt optimization affects translation quality, this study employed descriptive statistical methods. Descriptive statistics were used to summarize and interpret the experimental data, revealing overall trends in translation quality scores. However, it is important to recognize that descriptive statistics do not allow for significance testing. Consequently, the study’s conclusions focus on illustrating general trends rather than confirming the statistical significance of prompt optimization. Future research could incorporate more rigorous statistical techniques, such as paired t-tests, to assess the significance of the differences in translation quality before and after prompt optimization.

## 5: Linguistic theory

### 5.1: Translation patterns of Japanese "nominative" inner relation attributive clauses

When translating Japanese "nominative" inner relation attributive clauses into Chinese, the decision to adopt the "splitting long attributive clause and main clause into two separate sentences" translation method poses a challenge in determining whether the modified noun should initially fill in the valence complement of the verb in the attributive clause or the valence complement of the verb in the main clause. Prioritizing the valence complement of the verb in the attributive clause will result in the content of the attributive clause appearing before the main clause, while prioritizing the valence complement of the verb in the main clause will make the content of the main clause appear before that of the attributive clause.

If the modified noun gives priority to filling in the valence complement of the verb in the attributive clause, then the translation pattern is:

**Pattern I:** (Inner relation attributive clause + modified noun) & Main clause (Japanese) = > Attributive clause & modified noun, main clause (Chinese)

If the modified noun gives priority to filling in the valence complement of the verb in the main clause, then the translation pattern is:

**Pattern II:** (Inner relation attributive clause + modified noun) & Main clause (Japanese) = > Modified noun & main clause, Inner relation attributive clause (Chinese)

In (11), the translated sentence of (10), the modified noun "平介 (Heisuke)" gives priority to filling in the valence complement of "帰宅した (came home)", which is the verb in the attributive clause. The content of this attributive clause "この日夜勤明けで、午前八時ちょうどに帰宅した (came home from his night shift at exactly 8 a.m.)" comes before the main clause "四畳半の和室に入るなり、テレビのスイッチを入れた (entered the small tatami room, and turned on the television)". On the other hand, in (12), "平介 (Heisuke)", the modified noun, gives priority to filling in the valence complements of the verbs "入る (entered)" and "入れた (turned on)", which are verbs in the main clause. The content of the main clause comes before that of the attributive clause.

(10)この日夜勤明けで、午前八時ちょうどに帰宅した平介は、四畳半の和室に入るなり、テレビのスイッチを入れた。(Heisuke came home from his night shift at exactly 8 a.m., entered the small tatami room, and turned on the television)

(秘密(Naoko: A Novel [33]))

(11)平介这一天下夜班, 回到家刚好是上午八点, 一走进四叠大小的和室, 便打开了电视。

(Pattern I, Translated by the author)

(12)平介一走进四叠大小的和室, 便打开了电视。他这一天值完夜班, 回到家刚好是早上八点。

(Pattern II, Translated by the author)

### 5.2: Exploring the relationship between the semantic role of modified nouns and translation pattern selection

How should we decide whether the modified noun should be placed in the valence complement of the attributive clause verb or the valence complement of the main clause verb? We propose a hypothesis: the priority order depends on the strength of the subordinate relationship between the semantic role of the modified noun and the verb. If the modified noun’s semantic role has a stronger subordinate relationship with the attributive clause verb, it should be placed first in the valence complement of the attributive clause verb. Conversely, if the modified noun’s semantic role has a stronger subordinate relationship with the main clause verb, it should be placed first in the valence complement of the main clause verb. If there is no difference in the strength of the subordinate relationship between the modified noun and the main clause verb and that with the attributive clause verb, the modified noun can be placed in either valence complement first.

**Fig 2:**
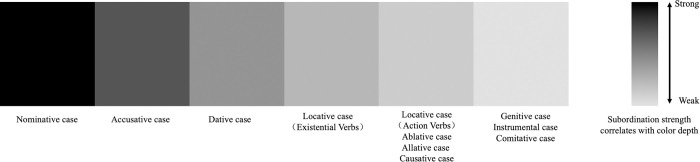
Degree of subordination of verbs. Note: Fig 2 was compiled by the author based on [34]’s research.

[34] proposed that Japanese language represents various subordinate relationships between nouns and verbs with different semantic roles. Table 1, drawn from Nitta’s research, lists the strong and weak subordinate relationships between each semantic role and verb. In this study, we will employ this theory to examine and evaluate the strength of the relationship according to the classifications in the Fig 2.

**Table 1:** Semantic role relationship between modified noun and attributive/main clause verb in inner relation attributive clauses.

		semantic role relationship between modified noun and main clause verb	Pattern II	Pattern I	Others	Total
**semantic role relationship between modified noun and attributive clause Verb**	Nominative case	Nominative case	3	21	0	24
		Accusative case	0	7	14	21
		Dative case	2	9	0	11
		Causative case	0	4	0	4
		Allative case	0	1	0	1
		Locative case	0	1	0	1
		Adverbial	0	2	0	2
**Total**			5	45	14	64

To validate the aforementioned hypothesis, this paper extracted 64 instances of data pertaining to "nominative" inner relation attributive clauses from five Japanese novels and examined the translation patterns of their respective Chinese versions. The analysis results have been summarized in Table 1.

When the semantic role relationship between the modified noun and the verb in the main clause is “nominative,” the strength of the subordinate relationship between the semantic role of the modified noun and the verb in the main clause and the subordinate relationship between the semantic role of the modified noun and the verb in the attributive clause are equal. It is abbreviated as “attributive clause verb = main clause verb.” When the semantic role relationship between the modified noun and the verb in the main clause is other than nominative, then the subordinate relationship between the semantic role of the modified noun and the verb in the main clause is weaker than that between the semantic role of the modified noun and the verb in the attributive clause. It is abbreviated as “attributive clause verb > main clause verb.” The correlation between the degree of subordination between semantic roles attributed to modified nouns and verbs and the selection of translation mode is presented in Table 2.

**Table 2:** Degree of subordination between attributive/main clause verbs.

	attributive clause verb > main clause verb	attributive clause verb = main clause verb
**Pattern I**	24 (60%)	21 (87.5%)
**Pattern II**	2 (5%)	3 (12.5%)
**Others**	14 (35%)	0 (%)
**Total**	40 (100.00%)	24 (100.00%)

Based on the results presented in Table 2, it is evident that Pattern I has a significantly higher number of translations than Pattern II. This supports the previously proposed hypothesis that the choice of translation mode for "nominative" inner relation attributive clauses is linked to the degree of subordination between the semantic role of the modified noun and the verb. As "nominative" inner relation attributive clauses have a "nominative" semantic relationship, which represents the highest level of subordinate attention intensity, when translating into Chinese, it is recommended that priority be given to using the modified noun as the valence complement of the attributive clause verb–nominative.

## 6: Prompt-enhanced machine translation

Section 5 of this paper yielded a crucial finding regarding the translation of Japanese "nominative" inner relation attributive clauses into Chinese. The study concluded that priority should be given to using the modified noun to fill the nominative case of the attributive clause verb and placing the content of the attributive clause before the content of the main clause to ensure efficient translation. In this section, we incorporate this linguistic insight into the prompt design for LLMs to assess whether a general prompt chaining, enriched with linguistics theory, can enhance the translation quality produced by these models.

### 6.1: Machine translation linguistic metric

To accurately assess the quality of machine translation, it is essential to establish comprehensive evaluation criteria. Building on [35] Japanese-English machine translation evaluation framework, we have developed Japanese-Chinese machine translation evaluation criteria that take into account the unique characteristics of Japanese-Chinese translation. The resulting evaluation framework is presented in Table 3 for a more intuitive and informative reflection of machine translation quality.

**Table 3:** Japanese-Chinese machine translation quality evaluation criteria.

Score 5	The original information is complete and accurate, with no omissions or mistranslations, and no grammatical errors. The translated content is natural and fluent and is identical to the original content. Native Chinese speakers can easily understand the translation and obtain semantic information that is essentially the same as in the original text.
Score 4	The original information is error-free, with no omissions or mistranslations, and no grammatical errors. The translated content closely resembles the original content in meaning and is also clear and natural. Native Chinese speakers can easily understand the translation and obtain semantic information that is essentially the same as in the original text.
Score 3	The original information is largely complete and accurate, with few omissions or mistranslations, and few or no grammatical errors. The translated content is similar to the original content in meaning and is mostly clear and easy to understand. Native Chinese speakers can comprehend the translation with relative ease and obtain most of the semantic information in the original text. However, the inclusion of attributive clauses may make the translation feel somewhat unnatural or less fluent to native Chinese speakers.
Score 2	The original information contains serious omissions or mistranslations, or major grammatical errors. The translated content significantly differs from the original content in meaning and is difficult to understand. Native Chinese speakers can comprehend the translation with difficulty but cannot obtain the same semantic information as in the original text.
Score 1	The translation is incomprehensible to native Chinese speakers and fails to convey the intended meaning of the original text.

### 6.2: Experiments and findings

Although the linguistic conclusion is valuable in enhancing translation quality, it may not be immediately applicable to improve the output of translation websites like Google and Baidu. It’s because these websites operate solely in the "input-output" mode, with no provision for users to debug the model. Fortunately, LLMs like ChatGPT are interactive translation tools that function within a dialogue-based framework, allowing users to iteratively obtain superior outputs by adding prompts. This paper aims to convert the linguistic conclusion derived in Section 5 into a prompt and evaluate its effectiveness by comparing the translation quality before and after implementing the prompt.

The recommendation for translating "nominative" inner relation attributive clauses into Chinese is to prioritize using the modified nouns to fill the valence complement of the attributive clause verbs, which means filling the nominative case of the attributive clause verbs with the modified nouns. Additionally, "the content of the attributive clause is placed before the content of the main clause" can be transformed into three-step prompt chaining:

Prompt A: What is the noun modified by the attributive clause in the following sentence? "SL".

Prompt B: Please place the noun modified by the attributive clause in the subject position of the attributive clause, and then separate "SL" into two sentences.

Prompt C: Translate the following sentence into Chinese. " SL‴

These three prompts—Prompt A, Prompt B, and Prompt C—form a chain in which "SL" refers to the original Japanese text in Prompts A and B, while "SL‴ in Prompt C is the modified Japanese text that has been split into two sentences based on the LLM’s response to Prompt B.

To assess the efficacy of the prompt, we selected five corpora featuring Japanese "nominative" inner relation attributive clauses and conducted translation experiments using ChatGPT. The experimental procedure entailed the following steps (Table 4).

**Table 4:** Experimental procedure for assessing prompt efficacy on Japanese inner relation attributive clauses.

User	(Q1:) Question: Translate the following sentence into Chinese: 社員の真似をして、勢いよくお辞儀をした私に、女性は笑って「ありがとうね、またきます」と言い、レジから去って行った^a^。
ChatGPT	(A1:) Answer^b^: 看到店员的样子, 我也效仿着热情地鞠了一躬, 然后那位女性笑着对我说: “谢谢啦, 下次还会再来的。”她说完后离开了收银台^c^。
User	(Q2:) Question: What is the noun modified by the attributive clause in the following sentence? 社員の真似をして、勢いよくお辞儀をした私に、女性は笑って「ありがとうね、またきます」と言い、レジから去って行った。
ChatGPT	(A2:) Answer: The noun that is modified by the attributive clause in this sentence is 私 (I).
User	(Q3:) Question: Please place the noun modified by the attributive clause in the subject position of the attributive clause, and then separate "社員の真似をして、勢いよくお辞儀をした私に、女性は笑って「ありがとうね、またきます」と言い、レジから去って行った" into two sentences.
ChatGPT	(A3:) Answer: 私は社員の真似をして、勢いよくお辞儀をした。女性は私に笑って「ありがとうね、またきます」と言い、レジから去って行った^d^。
User	(Q4:) Question: Translate the following sentence into Chinese. 私は社員の真似をして、勢いよくお辞儀をした。女性は私に笑って「ありがとうね、またきます」と言い、レジから去って行った。
ChatGPT	(A4:) Answer: 我模仿店员的样子, 热情地鞠躬。女性笑着对我说: “谢谢你, 再见”, 然后离开了收银台^e^。

^a^ The SL sentence(Q1) is from the novel コンビニ人間 (CONVENIENCE STORE WOMAN). The English translation of this section is " ’ Thank you! ’ I said, enthusiastically bowing the way the manager had done. The woman laughed and said, ’Thank you, I’ll come again, ’ and moved away from the till.". Translated by Kerim Yasar.

^b^ The machine translation site utilized in this study generated all translated texts, which were obtained on March 26, 2023.

^c^ The English translation of A1 is "Seeing the appearance of the store clerk, I also imitated and bowed enthusiastically. Then, the woman smiled at me and said, ’Thank you, I’ll come again next time.’ After saying that, she left the cashier counter." Translated by the author.

^d^ The English translation of A3 is "I imitated the employee, bowed energetically. The woman smiled at me and said, ’Thank you, I’ll come again,’ then left the cashier". Translated by the author.

^e^ The English translation of A4 is "I imitated the appearance of the store clerk, bowed enthusiastically. The woman smiled and said to me, ’Thank you, goodbye,’ then left the cashier counter". Translated by the author.

By adding a prompt, the Japanese translation of "社員の真似をして、勢いよくお辞儀をした私に、女性は笑って「ありがとうね、またきます」と言い、レジから去って行った"changed from "看到店员的样子, 我也效仿着热情地鞠了一躬, 然后那位女性笑着对我说: “谢谢啦, 下次还会再来的。” 她说完后离开了收银台" (A1) to "我模仿店员的样子, 热情地鞠躬。女性笑着对我说: “谢谢你, 再见”, 然后离开了收银台" (A4). The translation quality has also been improved from 4 to 5.

**Fig 3:**
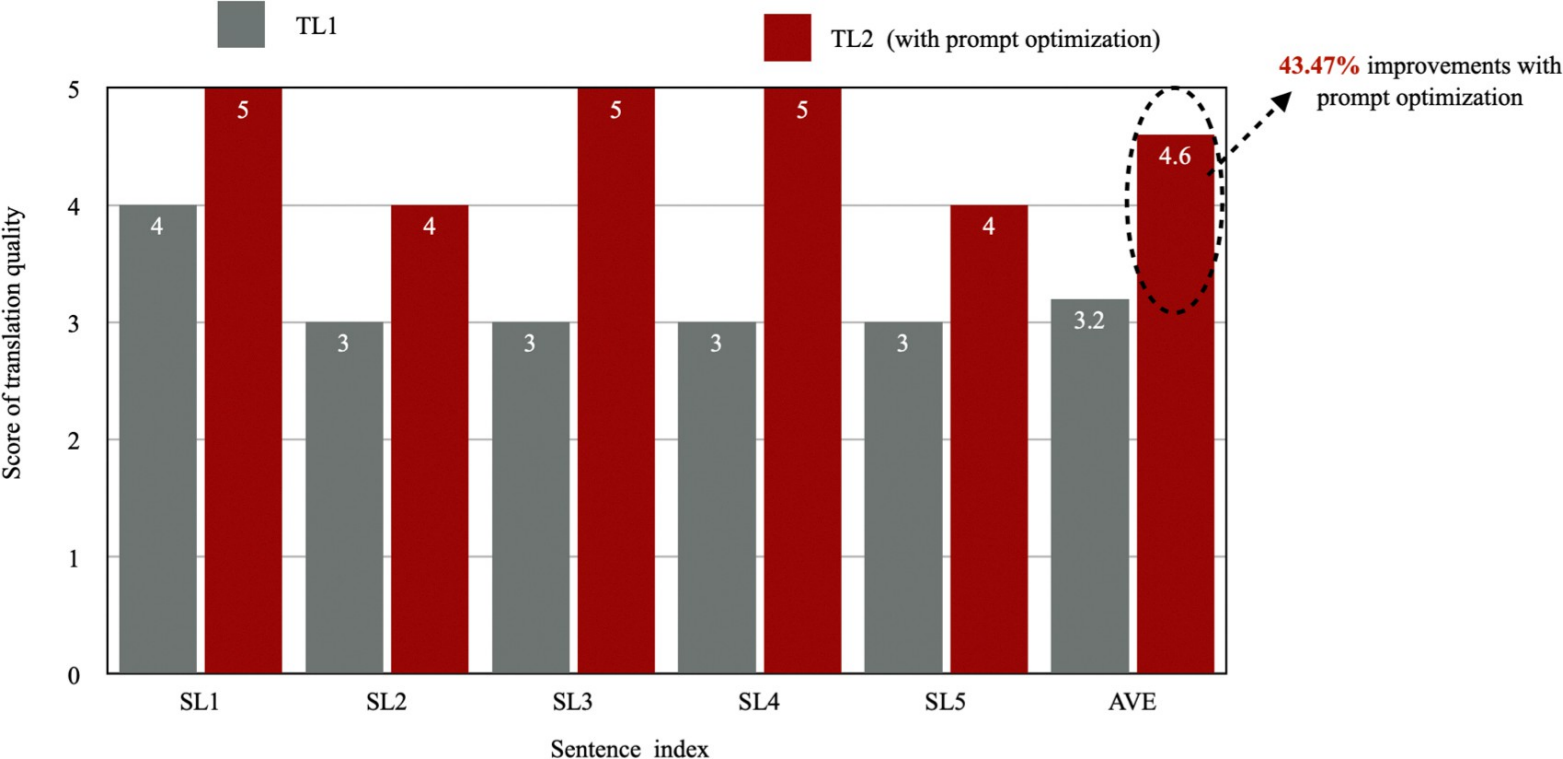
Translation quality changes before and after prompt optimization (translation quality score).

This paper conducted the above experiments on five data samples. We performed the same experiment on the remaining four Japanese texts. The translation produced by Result A1 corresponding to Prompt Q1 is referred to as TL1, and the translation generated by the final Result A4 corresponding to the adjusted prompt combination is referred to as TL2. Table 5 below presents TL1 and TL2 for each text. Fig 2 displays the translation quality scores for TL1 and TL2 for each set of SL. Fig 3 illustrates the translation method choices for TL1 and TL2 for each set of SL.

**Table 5:** Translation quality changes before and after prompt optimization. (SL and TL).

Source Language (SL^a^)	ChatGPT^b^	
	Target Language1 (TL1) (without prompt optimization)	Target Language 2 (TL2) (with prompt optimization)
1.社員の真似をして、勢いよくお辞儀をした私に、女性は笑って「ありがとうね、またきます」と言い、レジから去って行った。(’ Thank you! ’ I said, enthusiastically bowing the way the manager had done. The woman laughed and said, ’Thank you, I’ll come again, ’ and moved away from the till.)	看到店员的样子, 我也效仿着热情地鞠了一躬, 然后那位女性笑着对我说: “谢谢啦, 下次还会再来的。”她说完后离开了收银台。	我模仿店员的样子, 热情地鞠躬。女性笑着对我说: “谢谢你, 再见”, 然后离开了收银台。
2.駅前のショッピングモールで買ったケーキを食べながら、皆の顔を見て懐かしい懐かしいと連呼するユカリに皆が笑った。(We all laughed as she kept looking around, saying how much she’d missed us as we nibbled at the cakes from the station mall.)	一边吃着在车站前购买的蛋糕, 一边看着大家的脸, 不停地喊着“好怀念, 好怀念”的Yukari让大家都笑了。	当Yukari一边吃着她在车站前的购物中心买的蛋糕, 一边看着每个人的脸并不停地说着“好怀念, 好怀念”的时候, 大家都笑了。
3.詳しい事情も聞かずに突然祝福し始めた妹に、少し困惑した。(“Really?” I was a bit taken aback by her congratulating me without even asking for any more details.)	不问详细情况就突然开始祝福的妹妹, 让我感到有些困惑。	妹妹在不问详细情况的情况下突然开始祝福。我感到有些困惑。
4.身につけている洋服も、発する言葉のリズムも変わってしまった私が笑っている。(It was the me with different clothes and speech rhythms that was smiling.)	身上穿着的衣服和说话的语调都变了的我, 却在笑着。	我身上的衣服和说话的节奏都变了, 但我还是在笑。
5. ぐにゃりと首を曲げて目を閉じている小鳥を囲んで、他の子供たちは泣いていた。(It lay there with its neck twisted and eyes closed, and the other children were all standing around it crying.)	其他孩子们围绕着一只弯曲着脖子闭着眼睛的小鸟, 哭了起来。	小鸟弯着脖子闭着眼睛, 其他孩子们围着它哭泣。

^a^ SL sentences are from the novel コンビニ人間 (CONVENIENCE STORE WOMAN). The English translations of SL sentences are translated by Kerim Yasar.

^b^ The machine translation site utilized in this study generated all translated texts, which were obtained on March 26, 2023.

In Fig 3, the X-axis represents translation quality scores, and the Y-axis represents the five SL sets. The blue bars indicate the translation quality scores before optimization (TL1), while the orange bars represent the scores after optimization (TL2).

From Table 5 and Fig 3, it is evident that the quality scores of all five translations (TL2) after prompt optimization are higher than those produced using the zero-shot prompt (TL1). Specifically, by integrating linguistic prompts into ChatGPT, the average translation quality improved significantly from 3.2 to 4.6, marking a notable 43.75% increase. This demonstrates that designing prompts based on linguistics theory can indeed enhance the quality of Chinese translations of Japanese sentences with complex attributive clause structures.

Moreover, (13), (14), and (15) illustrate that TL1 incorporated linguistic details absent in SL, such as "I saw the behaviour of the clerk," whereas TL2 reflected content akin to that of SL, showing no signs of over-translation or under-translation, or mistranslation. Additionally, TL2 features a subject placement consistent with Chinese expression norms, rendering the language more natural, fluent, and concise compared to TL1.

(13)社員の真似をして、勢いよくお辞儀をした私に、女性は笑って「ありがとうね、またきます」と言い、レジから去って行った。

(SL)

(14)看到店员的样子, 我也效仿着热情地鞠了一躬, 然后那位女性笑着对我说: “谢谢啦, 下次还会再来的。”她说完后离开了收银台。

(TL1)

(15)我模仿店员的样子, 热情地鞠躬。女性笑着对我说: “谢谢你, 再见”, 然后离开了收银台。

(TL2)

**Fig 4:**
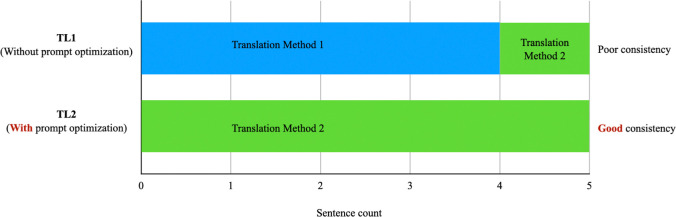
Translation method changes before and after prompt optimization.

In Fig 4, the blue bars represent the translation method that directly translates the Japanese "attributive clause + modified noun" structure into the corresponding Chinese structure (Translation Method 1), while the orange bars represent the method that does not directly translate this structure (Translation Method 2). The X-axis shows the translations generated using the zero-shot prompt (TL1) and those generated after prompt optimization (TL2), while the Y-axis indicates the frequency of each translation method.

From Table 5 and Fig 4, we observe that with the zero-shot prompt, one set of SL (SL1) used Translation Method 1, while four sets of SL (SL2, SL3, SL4, SL5) employed Translation Method 2. However, after applying the optimized prompt, all five sets of SL adopted Translation Method 1. This demonstrates that, with the optimized prompt, the large language model exhibits greater consistency in its choice of translation strategies. This consistency further suggests that incorporating linguistics theory into prompt design ensures greater stability in translation quality when large language models process Japanese sentences with complex attributive clause structures.

## 7: Discussion

In Section 1, the research objectives were outlined as follows: (1) to explore whether linguistics theory can be used to design prompts that enhance the quality of Chinese translations for Japanese sentences with complex "attributive clause + modified noun" structures, and (2) to determine whether linguistics theory can be utilized to design prompts that ensure the stability of translation quality when LLMs handle such structures. The experimental results presented in Chapter 6, including Table 5, Figs 3, and 4, show that integrating linguistics theory into prompt design significantly enhanced translation quality, with scores increasing from 3.2 to 4.6—a 43.75% improvement. Additionally, the large language model demonstrated greater consistency and stability in its selection of translation strategies. These findings provide strong evidence for the effectiveness of linguistically informed prompt design in practical machine translation applications, particularly in improving translation quality for Japanese-Chinese translations that involving "attributive clause + modified noun" structures.

### Practical applications

1. **Meeting user demand for high-quality translations in machine translation tools**: By integrating linguistics theory into prompt design, this study optimized prompt combinations, resulting in translations that are more natural and fluent when large language models handle Japanese-to-Chinese translation tasks involving complex "attributive clause + modified noun" structures. These optimized translations reduce instances of mistranslation and over-translation, allowing users to access more accurate, high-quality translations, thereby minimizing the cognitive effort required to comprehend machine-generated texts.
2. **Enhancing human-machine collaborative translation efficiency**: In human-machine translation workflows, the quality of machine translations directly impacts the amount of post-editing required by human translators. The findings of this study significantly improve the quality of translations for Japanese-to-Chinese tasks, particularly for sentences with "attributive clause + modified noun" structures. This improvement reduces the time and effort required to correct errors such as omissions, mistranslations, and awkward sentence constructions. Consequently, human translators can focus more on higher-level tasks, such as refining linguistic style, ultimately enhancing overall translation efficiency.
3. **Supporting Japanese learners in mastering complex sentence structures and improving language education**: The optimized prompt combinations can be valuable tools for intermediate and beginner-level learners of Japanese, helping them better understand complex sentence structures. This not only aids learners in grasping the nuances of Japanese grammar but also enhances the effectiveness of language education, contributing to improved learning outcomes.

### Theoretical contributions

This study highlights the immense potential of combining linguistics theory with large language models in machine translation. This approach not only improves translation quality but also introduces an innovative framework for addressing the interpretability of large language models, often referred to as "black boxes." The proposed framework offers new insights into how machines process multilayered linguistic structures, providing a novel avenue for research in both Japanese linguistics and Japanese-Chinese translation studies.

Unlike previous studies, which either focused solely on linguistic or translation theory or explored how to improve large language models through the optimization of training data without incorporating linguistics theory or providing interpretability, this research uniquely combines linguistics theory with prompt design for large language models. This innovative approach not only results in significant improvements in translation quality but also introduces interpretability into machine translation systems, offering a fresh and valuable contribution to the field.

## 8: Conclusion

In this paper, we propose a new approach for enhancing the accuracy of machine translation models through the combination of linguistics theory and neural methods. By establishing a clear link between the semantic roles of modified nouns and the selection of translation modes in Japanese attributive clauses, we present new insights into the translation process and underscore the importance of incorporating linguistic cues to improve translation accuracy. Leveraging this linguistics theory, we generate prompts that are specifically designed to aid machine translation and conduct experiments utilizing ChatGPT to verify the practicality of our approach.

The experimental results support two key conclusions:

1. Linguistically informed prompt chaining can significantly enhance the quality of Chinese translations generated by large language models when handling complex Japanese sentence structures involving attributive clauses.
2. Prompt chaining designed with linguistics theory ensure greater robustness and consistency in translation quality when LLMs process complex Japanese sentences with attributive clauses.

This research provides a fresh perspective on the integration of translation theory with machine learning technologies, promoting a stronger intersection between linguistics and computer science.

However, there are certain limitations to this study. The study primarily focused on designing prompts for a specific type of Japanese attributive clause with an agentive internal relation, which may limit its applicability to other clause types with different structures. Additionally, the validation was based on a small sample and conducted only with ChatGPT, leaving room for further exploration with larger datasets and comparisons to other models like Google Gemini.

Future research will address these limitations by expanding prompt design to other types of attributive clauses and expanding the corpus and conducting multi-model validation. By addressing these limitations, future research will deepen our understanding of the role of prompt design in machine translation, facilitating its application to more complex linguistic structures and across diverse language pairs.

## Supporting information

S1 File (PDF)
