# Supplementary material for: Linguistically informed ChatGPT prompts to enhance Japanese-Chinese machine translation: A case study on attributive clauses
Source: PLoS One. 2025 Jan 9;20(1):e0313264. doi: 10.1371/journal.pone.0313264 (PMC11717246; doi:10.1371/journal.pone.0313264)
Supplement: S1 File — (PDF) [file pone.0313264.s001.pdf]

		原文	訳文	N	主N	ーン
1	翼ある闇	ソファーから身を起こした辻村が、慇いん懃ぎんに訊ねる。	“您是？”辻村欠身离开沙发，态度相当恭敬。	動作主	動作主	Pattern II
2	翼ある闇6	この蒼鴉城には畝傍のような化け物ばかりが棲息しているのでは、と恐れていた私は、萱彦を見て少し安心した。	我原本担心栖居苍鸦城的会不会净是像畝傍那样的怪物，如今见到萱彦心里稍稍松了一口气。	経験者	動作主・経験者	Pattern II
3	半落ち	今現在、体内のグランドキソンを吸収する薬を使い、下剤と合わせて、排出を試みているという。	对方汇报说高野贡已经恢复了意识，现在正在用药物吸收体 内的除草剂，并结合泻药来促进排出，不久就要进人血液透析阶段。	動作主	対象	IV
4	半落ち	電話を切った志木は短い息を吐いた。	志木挂断电话，叹了一又气。	動作主	動作主	Pattern II
5	半落ち	その夕刊を見たW地検の佐瀬検事が電話を寄越した。	W地方检察厅的佐濑检事打来了电话。大概是看过晚报才知道空白两天的事，所以刨根问底地询问情况。	動作主	動作主	PatternI
6	半落ち	その目を止め、メモを取る手を動かす。	只见他停下目光，动了动手记笔记，拿起文件中附的照片看看，然后再次把目 光移回格纸上。	動作主	対象	IV
7	半落ち	園長は、月に一度か二度、学校帰りの少女に寄り添って歩く派手な服装の女を目撃していた。	可是，园长却说每 个月总有一两次看到有个打扮入时的女人陪伴在放学回来的女孩身旁。	動作主	対象	IV
8	半落ち	検事宅への夜回りは禁止と知りながら、破れかぶれでぶつかってくる者もいる。	明知夜访检事住处是被禁止的，可 还是有些记者不管不顾地前来。	動作主	対象	IV

9	半落ち	複雑な事件を単純にしている者がいる。	有人把复杂的案子简单化了	動作主	対象	IV
10	半落ち	喉まで出かかった言葉を中尾は呑み込んだ。	话到了嗓子眼中尾又咽了下去	動作主	対象	Pattern II
11	半落ち	振り向くと、送話口を手で押さえた岩村が、深みのある視線を中尾に向けていた。	一回头，用手捂住话筒的岩村正用深邃的目光望着他。	動作主	動作主	Pattern II
12	半落ち	県警本部を後にした中尾は、県道を西に向けて車を走らせた。	中尾离开县警本部，沿县道驱车往西而去	動作主	動作主	Pattern II
13	半落ち	酔っているからといって簡単にネタを洩らす検事は存在しない。	就算是醉了，也没有一个检事会轻易透露情报。	動作主	対象	IV
14	半落ち	「なぜ認めないんです？　おかしいじゃないですか。たった半日前にあれほど怒っていたあなたが、なぜ筋を通せないんです？」佐瀬の目が力を失った。	“ 为什么不承认?真是可笑。就在半天前你还是那么义愤填膺， 可现在为什么就不通情理了呢?” 佐濑的目光已黯然失色。	経験者	経験者	Pattern II
15	半落ち	礼の言葉もそこそこに、毛皮のコートを抱えた痩やせぎすのワンピースはヒールを鳴らして部屋を出ていった。	致谢的话只有草草几句，然后，她手抱皮大衣，摇晃着连衣 裙下的干瘦身躯，踩着高跟鞋嗒嗒嗒地出了屋，压根儿就没有一丝愿意舍弃医生夫人宝座的意思。	動作主	動作主	Pattern II
16	半落ち	植村に誘いの声を掛ける同業者はいなかった。	同行中没有 一个人对植村发出邀请。	動作主	対象	IV
17	半落ち	一人だけ、上半身を起こしてこちらを見ている老人がいた。	只有一位老人直着上半身望着这边，没有焦点的凹陷的眼睛仿佛 在凝视着遥远的过去或者来世。	動作主	対象	IV
18	半落ち	腰を浮かせた植村を、まあ待てよと佐瀬が引き止めた。	植村刚要站起来，“ 等一下。” 佐濑又叫住了他。	動作主	対象	Pattern II
19	半落ち	地検を出た植村はバスで事務所に向かった。	ない	動作主	動作主	Pattern II

20	コンビニ人間	再びおにぎりを並べに走ろうとした私に、バイトリーダーの泉さんが声をかける。	正当我想跑去继续排放饭团的时候，兼职领班泉小姐向我问道：	動作主	相手	Pattern II
21	コンビニ人間9	ぐにゃりと首を曲げて目を閉じている小鳥を囲んで、他の子供たちは泣いていた。	小鸟的脖子软绵绵地歪着，双眼紧闭，围在它身边的其他孩子都哭了。	動作主	対象	Pattern II
22	コンビニ人間	私の頭を撫でて優しく言った母に、私は、「これ、食べよう」と言った。	母亲抚摩着我的头，温柔地说道。而我却说："把它吃了吧。"	動作主	相手	Pattern II
23	コンビニ人間	走ってきて、惨状を見た先生たちは仰ぎょう天てんし、私に説明を求めた。	老师刚跑过来，见到这惨状，惊得瞠目结舌，让我给出解释。	動作主	動作主	Pattern II
24	コンビニ人間	先生が何を怒っているのかわからなかった私はそう丁寧に説明し、職員会議になって母が呼ばれた。	我不明白老师在生什么气，仔细地解释给他听，结果是母亲被叫去参加教职员会议了。	経験者	動作主	Pattern II
25	コンビニ人間10	なぜだか深刻な表情で、「すみません、すみません」と先生に頭を下げている母を見て、自分のしたことはどうやらいけないことだったらしいと思った。	看到母亲表情严肃地一边说着"对不起，对不起……"一边向老师低头赔礼，我意识到自己的所作所为似乎是不对的。	動作主	対象	IV
26	コンビニ人間	必要なこと以外の言葉は喋しゃべらず、自分から行動しないようになった私を見て、大人はほっとしたようだった。	除非必要决不说话，从不做出自主的行动。看到这样的我，大人们似乎如释重负。	動作主	対象	IV
27	コンビニ人間	お菓子や玩おも具ちゃにあまり興味がなかった私は、それらを妹にあげることも多かった。	因为我对点心和玩具几乎不感兴趣，经常把那些东西让给妹妹，所以妹妹总爱围着我转。	経験者	動作主	Pattern II
28	コンビニ人間	研修で習っていない質問にまだうまく答えられない私を、社員が素早くフォローした。	培训中没有学过这种提问，我回答得支支吾吾的，职员迅速为我补救。	動作主	対象	Pattern II

29	コンビニ人間	社員の真似をして、勢いよくお辞じ儀ぎをした私に、女性は笑って「ありがとうね、またきます」と言い、レジから去って行った。	我模仿着职员，使劲鞠了个躬，妇人笑着说：“谢谢你，我下次还来。”便离开收银台走远了。	動作主	相手	Pattern II
30	コンビニ人間	いつまでも就職をしないで、執しつ拗ようといっていいほど同じ店でアルバイトをし続ける私に、家族はだんだんと不安になったようだが、そのころにはもう手遅れになっていた。	我迟迟不去找工作，还近乎偏执地只在同一家店继续做兼职，这似乎让家人逐渐担忧了起来，可那时已经为时已晚。	動作主	起因	Pattern II
31	コンビニ人間	私が手にしているマンゴーチョコレートのパンを目にした泉さんが言う。	泉小姐盯着我手中的杧果巧克力面包说道。	動作主	動作主	Pattern II
32	コンビニ人間	菅原さんの言葉を繰り返す私に、泉さんが時計と指輪を外しながら笑った。	看到我重复菅原小姐说的话，泉小姐一边摘下手表和戒指，一边笑了。	動作主	起因	Pattern II
33	コンビニ人間	明るく声をかけてきたミホと、持っているバッグが色違いだという話で盛り上がり、今度一緒に買い物に行こうと、メールアドレスを交換した。	爽朗地与我搭话的是美穗。只因为提的包是同款不同色，我们聊得热络起来，约定下次一起去购物，还交换了邮箱地址。	動作主	対称	Pattern II
34	コンビニ人間	駅前のショッピングモールで買ったケーキを食べながら、皆の顔を見て懐かしい懐かしいと連呼するユカリに皆が笑った。	由香里一边品尝着车站前购物中心买来的蛋糕，一边盯着大家的脸，连连说“好怀念好怀念”，大家都笑了。	動作主	起因	Pattern II
35	コンビニ人間	ぐっすりと眠るミホの子供を見つめるサツキを見ていると、二人の子宮も共鳴しあっているような気持ちになる。	看见皋月注视着由香里熟睡的孩子，我不禁觉得她们俩的子宫都在产生共鸣。	動作主	対象	IV

36	コンビニ人間	経性験はないものの、自分のセクシャリティを特に意識したこともない私は、性に無む頓とん着ちやくなだけで、特に悩んだことはなかったが、皆、私が苦しんでいるということを前提に話をどんどん進めている。	尽管没有性经验，我也没怎么在意过自己的性取向，只是对性事毫不在乎而已，并没有为此烦恼过。众人竟然以我在受苦为前提，一个劲地讨论这话题。	動作主	経験者	Pattern II
37	コンビニ人間	かごをレジに置く音が聞こえ、素早く振り向くと、つえをついた常連の女性客が立っていた。	购物篮放在收银台上的声音传来，我迅速回过头去，只见那位常来的妇人正撑着拐杖站在前方。	動作主	動作主	Pattern II
38	コンビニ人間	チョコレートを見ている女性に、「あー！駄目ですよ、せっかくきちんと並んでいるのにぐちゃぐちゃにして！」と叫んでいる。	看到有女顾客在挑选巧克力，也大叫道：“啊！这怎么行呢！人家好不容易摆得整整齐齐，你把它们弄得乱糟糟的！”	動作主	相手	Pattern II
39	コンビニ人間	私が言いつけたと思ったらしい白羽さんに、「いえ」と首を横に振ると、店長が言った。	白羽先生似乎以为是我告状的，我摇摇头说了句“没有”，店长就接着说了：	動作主	相手	Pattern II
40	コンビニ人間	今まで、近いからとか楽そうだからとか、いろいろな理由を聞いてきたが、そんな理由でコンビニで働き始めた人に会うのは初めてだった。	因为住得近所以很轻松之类的各种理由，我已经听过了不少，但还是第一次听说有人为这种理由来便利店上班的。	動作主	对称	PatternI
41	コンビニ人間	久しぶりに地元に帰ってきたという友達も何人かいたので、一人ずつ近況を言う流れになった。	有好几个朋友都是许久没回故乡了，于是一个个讲述起自己的近况来：	動作主	对象	IV

42	コンビニ人間	私を庇うようにミホが言う。私の代わりに言い訳をしてくれたミホに感謝していると、ユカリの旦那さんが、「え、でも立ち仕事でしょ？　身体弱いのに？」と怪訝な声を出した。	美穗袒护我似的说道。能替我说出借口，实在很感谢美穗。然而此时，由香里的丈夫却惊讶地问道：“咦？便利店不是整天站着吗？身体吃得消吗？”	動作主	相手	PatternI
43	コンビニ人間	店の外にいる白羽さんの姿に気が付いたのは偶然だった。	发现店外出现白羽先生的身影，完全是一次偶然。	動作主	非空間的着点	Pattern II
44	コンビニ人間	どちらかというと白羽さんが性犯罪者寸前の人間だと思っていたので、迷惑をかけられたアルバイト女性や女性客のことも考えずに、自分の苦しみの比喩として気軽に強姦という言葉を使う白羽さんを、被害者意識は強いのに、自分が加害者かもしれないとは考えない思考回路なんだなあ、と思って眺めた。	无论如何，白羽先生已经几乎是半个性犯罪者了，他丝毫没考虑过自己骚扰过的兼职女孩和女顾客，他竟然能把自己的痛苦随便比喻成“强奸”。受害者意识这么强，却丝毫没想过自己是个加害者。原来还有这种思路啊，我边想边打量他。	動作主	対象	Pattern II
45	コンビニ人間	え、自分の人生に干渉してくる人たちを嫌っているのに、わざわざ、その人たちに文句を言われないために生き方を選択するんですか？	咦？你明明讨厌别人干涉自己的人生，还偏要选择这种在乎他人看法的生活方式吗？	動作主	対象	IV
46	コンビニ人間	詳しい事情も聞かずに突然祝福し始めた妹に、少し困惑した。	还没了解详情，妹妹就突然开始祝福我了，让我有些困惑。	動作主	起因	Pattern II
47	コンビニ人間	出来立てのからあげ棒を並べていたトゥアンくんも、「からあげ棒、いかがデスカー！」と声を張り上げてくれた。	图安一边将刚做好的炸鸡串摆放上架，一边也嗓门大开地喊道。“炸鸡串特惠，欢迎选购！”	動作主	動作主	Pattern II

48	コンビニ人間	慌てて立ち上がった妹に、「別にそんなのしないでいいよ。ああ、でもそろそろ餌の時間かあ……」と言い、台所に置いてあった洗面器に、ご飯と、鍋の中にあるお湯で茹でられたじゃがいもとキャベツを入れ、風呂場に持って行った。	妹妹慌忙站起来。“不用打什么招呼的。啊，差不多到喂食的时间了……”我说着就取过厨房里的水盆，把米饭与锅中清煮的土豆和卷心菜装进去，带去了浴室。	動作主	相手	Pattern II
49	コンビニ人間	突然饒舌に喋り始めた白羽さんを、私は呆然と見上げた。	白羽先生突然变得能说会道，我目瞪口呆地仰望他。	動作主	対象	Pattern II
50	コンビニ人間	帰って早々、白羽さんの家庭の事情に巻き込まれた私は、ぐったりと疲れて白羽さんの話を聞く気にもなれず、	才刚到家，我就被卷进了白羽先生的家庭琐事之中，累得精疲力竭，根本没心情听白羽先生的话。	経験者	経験者	Pattern II
51	コンビニ人間	私の代わりに、レジでは先週から入ったミャンマー人の女の子がバーコードをスキャンしている。	收银台上，已经有上周刚进来的缅甸女孩代替我扫描条形码。	動作主	動作主	Pattern II
52	コンビニ人間	「何、馬鹿なことをやってるんだ、お前は！」<br>道路まで私を引き摺って怒鳴った白羽さんに、私は言った。<br>「コンビニの『声』が聞こえるんです」	“你在做什么蠢事呢！”白羽先生揪着我一直到路边，又怒喝道。<br>“我能听见便利店的‘声音’。”我回答。	動作主	相手	Pattern II
53	博士の愛した数式	友だちにからかわれるのを嫌がり、いつも帽子を被っていた息子は、警戒して首をすくめた。	儿子讨厌被伙伴们嘲笑，平常总戴着帽子，当下起了戒心，把小脑袋一缩。	動作主	動作主	Pattern II
54	博士	世界の成り立ちは数の言葉によって表現できると信じていた博士には、数えきれない、などという言い方は不快かもしれない。	博士相信世界的构成能够通过数字这种语言来表述，数不尽之类的说法可能会引起他的不快。	動作主	経験者	Pattern II

55	博士	学校でようやく分数を習ったばかりの息子は、博士の三十分足らずの説明でもう、ゼロより小さい数の存在を受け入れていた。	儿子在学校好不容易才学到分数，可听了博士不到三十分钟的课，就已经接受了比0更小的数字的存在。	動作主	動作主	Pattern II
56	博士	新しい家政婦だと告げた私に博士が一番に尋ねたのは、名前ではなく靴のサイズだった。	当我告诉他我是他的新保姆时，博士最先问的不是我的名字而是鞋子的尺码，	動作主	相手	Pattern II
57	博士	ひどい猫背のために一六〇センチほどしかない身長はますます小さく見え、骨張った首筋には皺の間に垢がたまり、ぱさついて好き勝手な方向に跳ねる白髪が、せっかくの福耳を半分覆い隠していた。	他佝偻得厉害，显得他估摸仅有160厘米的身体越发的瘦小；瘦骨嶙峋的脖颈上，皱纹之间积着体垢；一头白发乱蓬蓬的，随心所欲地跳向东跳向西，把一双难得的招福耳遮住了一半。	動作主	動作主	Pattern II
58	博士4	「あんな変人の義弟が寄生虫みたいにくっついて、旦那の遺産を食い潰しているんだから、あの未亡人もお気の毒だね」博士の数字攻撃に音を上げて一週間で首になった古参の家政婦が、しみじみと言った。	“有这么个怪人小叔子跟个寄生虫似的黏着甩也甩不掉，吃空她老公的遗产，那个寡妇也真是可怜哪！”一个老资格保姆无限感慨地说道。她招架不住博士的数字攻势，大叫吃不消，才一个礼拜就被辞退了。	経験者	動作主	PatternI
59	博士	これほどたくさん話し掛けてくれる博士を白けさせたくなくて、私は洗い物の手を止め、うなずいた。	博士难得主动跟我讲这么多话，我不愿败他的兴致，停下手上的活，点点头。	動作主	対象	Pattern II
60	秘密5	この日夜勤明けで、午前八時ちょうどに帰宅した平介は、四畳半の和室に入るなりテレビのスイッチを入れた。	这一天值完夜班，回到家刚好是早上八点。平介一走进四叠大小的和室，便打开了电视	動作主	動作主	PatternI

61	秘密	帰りが９時１０時になってしまう平介は、平日に妻の直子や娘の藻奈美と一緒に夕食を取ることができなかった。	平介经常回到家已经是晚上九十点钟，根本无法与妻子直子和女儿藻奈美一起共进晚餐。	動作主	動作主	Pattern II
62	秘密	せっかくの春休みというのに、仕事が忙しくてまともな家族サービスなどとてもしてやれそうにない平介としては、渡りに船といえなくもなかった。	难得女儿放了春假，平介却因工作的繁忙无法好好陪伴家人。因此对于平介来说，这未尝不是一次补偿的机会。	動作主	副詞的	Pattern II
63	秘密	たまたま直子の友人に、旅行代理店に勤めている女性がいたので、	凑巧的是，直子刚好有个朋友在旅行社工作，	動作主	対象	IV
64	秘密	二人だけで帰らせた私にも責任があります。	是我让她们母女俩回去的，我也有责任啊	動作主	間的位置	Pattern II
